# Symposium: evidence for the use of intra-articular cortisone or hyaluronic acid injection in the hip

**DOI:** 10.1093/jhps/hnv020

**Published:** 2015-03-31

**Authors:** Sivashankar Chandrasekaran, Parth Lodhia, Carlos Suarez-Ahedo, S. Pavan Vemula, Timothy J. Martin, Benjamin G. Domb

**Affiliations:** 1. American Hip Institute; 2. Hinsdale Orthopaedics

## Abstract

The primary purpose of this review article is to discuss the role of diagnostic, corticosteroid, hyaluronic acid (HA) and platelet rich plasma (PRP) in the treatment of osteoarthritis (OA) and femoroacetabular impingement (FIA). These treatments play an important biological role in the non-operative management of these conditions. Two independent reviewers performed an search of PubMed for articles that contained at least one of the following search terms pertaining to intra-articular hip injection—local anaesthetic, diagnostic, ultrasound, fluoroscopic, image guided, corticosteroid, HA, PRP, OA, labral tears and FAI. Seventy-two full text articles were suitable for inclusion. There were 18 articles addressing the efficacy of diagnostic intra-articular hip injections. With respect to efficacy in OA there were 25 articles pertaining to efficacy of corticosteroid, 22 of HA and 4 of PRP. There were three articles addressing the efficacy of biologics in FAI. Diagnostic intra-articular hip injections are sensitive and specific for differentiating between intra-articular, extra-articular and spinal causes of hip symptoms. Ultrasound and fluoroscopy improves the precision of intra-articular positioning of diagnostic injections. Corticosteroids are more effective than HA and PRP in alleviating pain from hip OA. A higher dose of corticosteroids produces a longer benefit but volume of injection has no significant effect. Intra-articular corticosteroids do not increase infection rates of subsequent arthroplasty. There is currently limited evidence to warrant the routine use of therapeutic injections in the management of labral tears and FIA.

## INTRODUCTION

Intra-articular and extra-articular injections around the hip joint are commonly used as both a diagnostic and therapeutic modality [1–3]. There have been discrepancies with respect to the efficacy of several of the biologics used in these injections [4–6]. These discrepancies exist despite the existence of level 1 double blinded randomized control trials. It is therefore important for the practicing orthopaedic surgeon to have current knowledge of the literature regarding the efficacy of many of these biologics so that patients may be informed appropriately about the risks and benefits of receiving injections around the hip joint.

Intra-articular injections of local anaesthetic into the hip joint has been shown to be a useful modality to ascertain an intra-articular pain trigger or identify the contribution of intra-articular pathology to a patient’s pain profile [7]. The utility of diagnostic hip injections has been particularly evident when differentiating between pain from hip and spinal pathology [8–10]. There have been several studies evaluating methods of delivery of diagnostic injections [11–14]. The benefit of non-image guided injections based on anatomical landmarks is the ability to perform them at initial consultation without the additional cost and required availability of ultrasound or fluoroscopy [14, 15], while disadvantages include reduced precision [16]. Ultrasound has the advantage of accurately localizing intra-articular placement of the injection, the ability of being performed at the time of initial consultation and no radiation as compared with fluoroscopy [16]. The disadvantage is the expense of the equipment and the training required to interpret imaging [17]. Fluoroscopic guided injections often need to be performed at a different patient encounter and expose the patient to radiation [16].

Corticosteroids, hyaluronic acid (HA) and platelet rich plasma (PRP) are the most common biologics used in intra-articular hip injections. Corticosteroids have been shown to be effective in reducing pain associated with osteoarthritis (OA) of the hip [18]. Important risk factors include local infection and soft tissue irritation at the site of injection, exacerbation of pain and septic arthritis [19]. Another important consideration is the *in* *vitro* chondrotoxicity of corticosteroids and local anaesthetics on human chondrocyte populations [20–21]. This toxicity has not been demonstrated *in* *vivo* but is an important consideration in any joint preserving intervention in the young adult hip population. Additional, factors that need to be considered when using intra-articular steroid injections include dose, frequency and proximity to any surgery as there is potential risk of increased post-operative infection rate [20]. There has been mixed results in the literature on the efficacy of HA and PRP in the treatment of pain due to OA of the hip [23]. Several studies have suggested a potential use in the non-operative treatment of OA but this role needs to be more clearly defined in respect to their use of other pathologies [24].

The purpose of this article is to provide a comprehensive review on the use of intra-articular injections in the hip. First, the article will aim to synthesize the literature and provide an algorithm for the indications, method of delivery and interpretation of diagnostic hip injections. Second, the article will review the efficacy of corticosteroids, HA and PRP in the non-operative treatment of OA; part of this will included a risk benefit analysis on the use of each of these biologics. Finally, the article will review the use of intra-articular injections in the management of labral tears and femoroacetabular impingement (FAI).

## METHODS

**Fig. 1.**
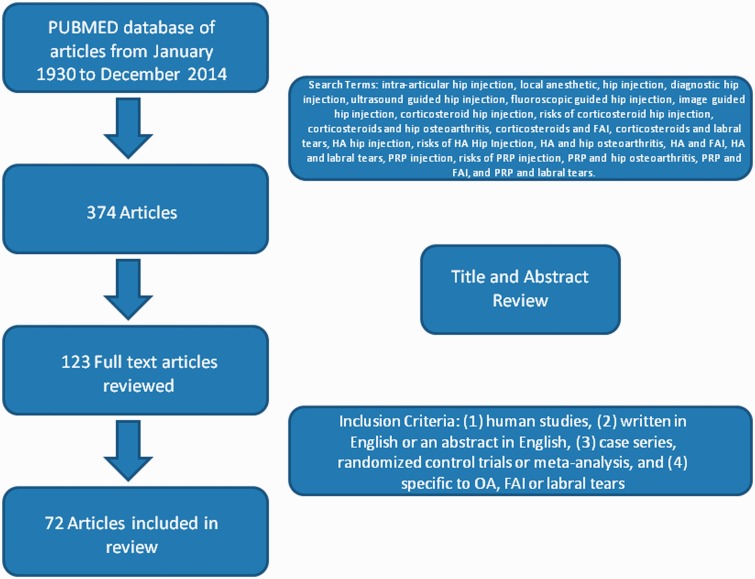
Flow chart on article selection to be included in this review.

Two independent reviewers (S.C. and P.L.) performed an extensive search of PubMed for articles that contained at least one of the following search terms: intra-articular hip injection, local anaesthetic hip injection, diagnostic hip injection, ultrasound guided hip injection, fluoroscopic guided hip injection, image guided hip injection, corticosteroid hip injections, risks of corticosteroid hip injection, corticosteroids and hip OA, corticosteroids and FAI, corticosteroids and labral tears, HA hip injections, risks of HA hip injection, HA and hip OA, HA and FAI, HA and labral tears, PRP hip injections, risks of PRP hip injection, PRP and hip OA, PRP and FAI and PRP and labral tears. The search included articles published from January 1930 to December 2014. Reference lists from relevant articles were also reviewed to identify any additional studies of interest. The search revealed 374 articles, 251 were excluded after title and abstract review. Based on initial review,123 full text publications were reviewed and 72 of these articles met our inclusion criteria (Fig. 1). These were (i) human studies, (ii) written in English or an abstract in English, (iii) case series, randomized control trials or meta-analysis and (iv) specific to OA, FAI or labral tears. Articles were excluded if they were review or technique articles, case reports, non-operative studies, or involved inflammatory arthritis, avascular necrosis, post-traumatic arthritis, Legg–Calves–Perthes disease and septic arthritis as the primary pathology.

## RESULTS

Using the above described search criteria, 72 articles ultimately met appropriate criteria for inclusion in this review. These articles were divided in the categories based on whether the focus was on diagnostic hip injections, the role of corticosteroid, HA and PRP intra-articular hip injections in OA and management of labral tears and FAI. Table I summaries the number of articles in each category and the level of evidence pertaining to these articles.

**Table I.** A summary of the number of articles and their level of evidence used in this review

Injection	Diagnostic	Corticosteroid in OA	HA in OA	PRP in OA	FAI
Randomized prospective studies	3	3	5	1	
Non-randomized prospective studies	7	10	8	1	3
Retrospective studies	8	13	9	2	
Total	18	26	22	4	3

## DIAGNOSTIC HIP INJECTIONS

### Efficacy of diagnostic local anaesthetic intra-articular hip injections

There have been several studies that have demonstrated that diagnostic local anaesthetic intra-articular hip injections are useful in differentiating between intra-articular and extra-articular sources of pain [8, 25–27]. Crawford *et al*. [25] investigated the utility of diagnostic intra-articular injections in 42 patients who were being considered for primary total hip arthroplasty (THA) but presented with an atypical pain profile for hip OA. Thirty-two out of 33 patients who reported relief of their symptoms from an intra-articular anaesthetic injection had a subsequent successful THA. The authors concluded that a diagnostic intra-articular injection was at least 96% sensitive for diagnosing an intra-articular source of pain. Similarly, Deshmukh *et al*. [8] in their retrospective review of 204 consecutive diagnostic hip injections demonstrated a sensitivity of 91.5%, specificity and positive predictive value of 100% and negative predictive value of 84.6% for a positive response proceeding a successful THA. Several other published series have demonstrated concordant results [7, 9, 27, 29].

Not only have diagnostic intra-articular hip injections been shown to predict successful outcomes after THA, they have also demonstrated utility in the differentiation of coxalgia from an intra-articular versus a neuropathic source. Kleiner *et al*. [9] reported a sensitivity and specificity of 87% and 100% respectively in a series of 18 patients undergoing diagnostic intra-articular hip injections to differentiate between intra-articular causes of hip pain from neurological ones. Pateder *et al*. [30] and Faraj *et al*. [31] also reported similar specificities and sensitivities for diagnostic hip injections in differentiating between hip and spinal pathology.

In contrast to the above findings, the results of diagnostic intra-articular hip injections have not been as useful in the management of hip dysplasia. Spruit *et al*. [32] conducted a double blinded randomized study of 40 patients with symptomatic hip dysplasia infiltrated with either bupivacaine or placebo. There was no significant difference between the groups for pain relief or duration of pain relief. They concluded a limited utility of diagnostic hip injections in predicting outcome after surgery in this patient cohort.

With respect to labral tears, Arnold *et al*. [33] found that a fluoroscopic guided intra-articular hip injection of local anaesthetic combined with a pain circle diagram may help physicians reconcile the expectations of those patients with labral tears who believe that hip arthroscopy will treat their multiple areas of ‘hip’ pain. Specifically, central groin pain and lateral peritrochanteric pain were more likely to relieved with a diagnostic hip injection and have a higher likelihood of improvement with labral surgery than ischial tuberosity and anterior thigh pain. The latter two areas were less likely to be relieved by intra-articular hip injections of local anaesthetic in the 52 patients evaluated in their study.

**Fig. 2.**
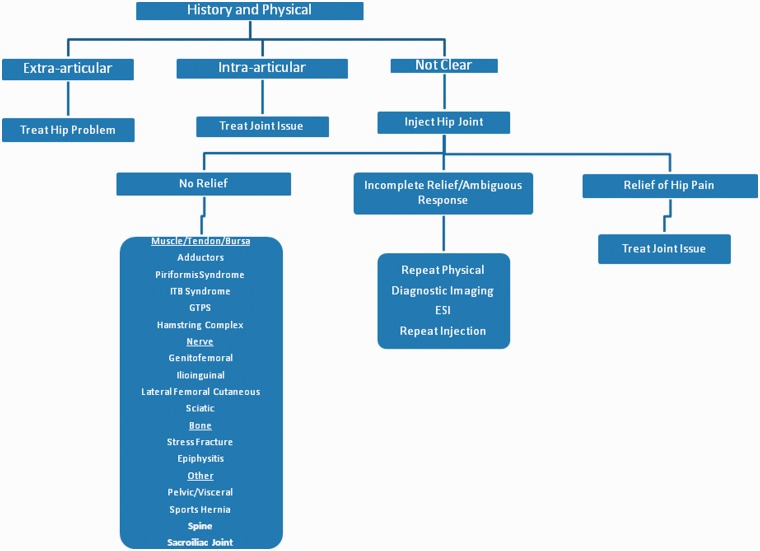
Algorithm for the evaluation of atypical hip symptoms.

In summary, diagnostic local anaesthetic intra-articular hip injections are beneficial in elucidating the significance of intra-articular pain generators and predicting outcomes following THA and labral surgery. Figure 2 provides an algorithm for the incorporation of diagnostic injections into the history and physical examination of patients with atypical hip symptoms. A positive response would be an indication to treat the hip joint problem whereas a negative response should prompt further investigation of extra-articular hip, spinal or sacroiliac joint pathology.

### The role of image guidance for intra-articular hip injections

Intra-articular hip injections may be localized using anatomical landmarks or image guidance. Anatomical landmarks rely on surface marking of a vertical from the anterior superior iliac spine and a horizontal 1 cm proximal to the midpoint of the greater trochanter [12, 14, 15, 34–38]. The needle entry point is commonly anterior or anterolateral with posterior angulation towards the hip joint base on radiographic evaluation of femoral neck valgus and anteversion [38]. Ultrasound and fluoroscopy are the imaging modalities most commonly used to facilitate intra-articular hip injections [11, 13, 17, 38, 39–42]. The main advantage of the non-image guided technique is the reduced expense associated with ultrasound and fluoroscopy [14]. The main disadvantage is the reduced precision of intra-articular needle placement [39].

**Fig. 3.**
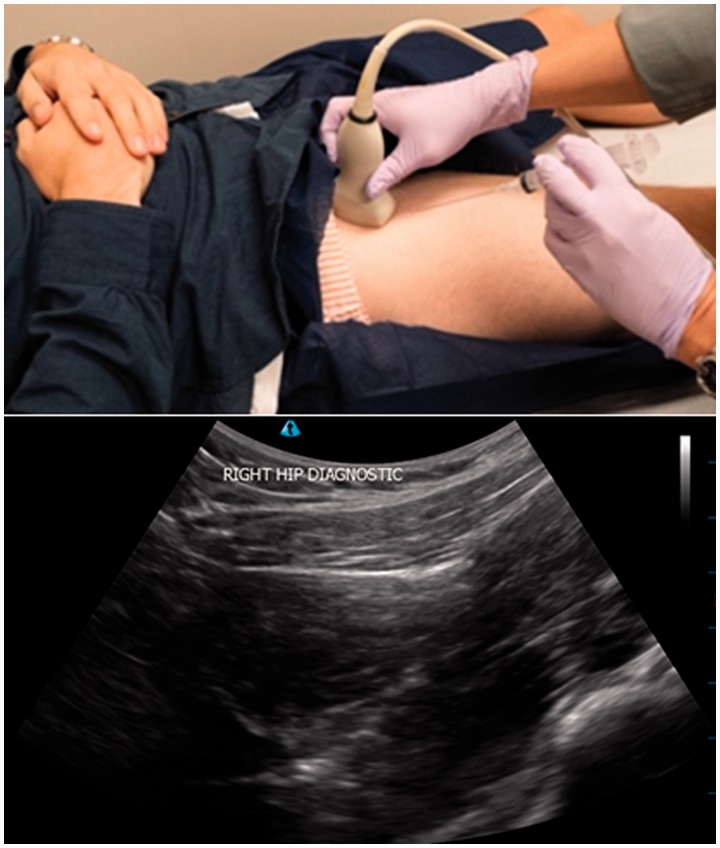
Protocol for intra-articular local anaesthetic injection for the hip: 10 ml of 1% lignocaine is inserted under ultrasound guidance. The patient is reviewed after 30 min for improvement of pain and impingement signs.

Several studies have shown that image guide intra-articular hip injections improves the accuracy of intra-articular placement [11, 12, 15, 35, 39, 40]. The accuracy of non-image guided injections range from 67% to 88% [12], which improves to 97% with the use of ultrasound [40]. Furthermore, ultrasound in contrast to fluoroscopy is not associated with radiation exposure. Ultrasound guided injections can also be given at the time of initial consultation. For this reason the authors employ ultrasound guided injections in their own practice and Fig. 3 is an outline of the technique used.

## CORTICOSTEROID INTRA-ARTICULAR HIP INJECTIONS

### The efficacy of corticosteroid intra-articular hip injections for pain relief for OA of the hip

Kullenberg *et al*. [19] conducted one of the largest double-blinded randomized controlled trials on the efficacy of intra-articular corticosteroid injection on pain relief in OA of the hip. They randomized 80 patients with pain for >4 weeks to 80 mg of triamcinolone acetonide or 1% mepivacine with 40 patients in each group. At 3- and 12-week follow-up there was a significant reduction in pain (more at weight bearing than at rest), increased joint range of motion and improved functional ability compared with the local anaesthetic group who had no improvement in these parameters. They concluded that intra-articular corticosteroids improve pain and functional range of motion in patients with hip OA. This conclusion has also been verified by several other studies but many of these were neither randomized nor blinded and hence are more prone to effects of bias [19, 43–49].

Robinson *et al*. [49] analysed not only the clinical effectiveness but also the dose response of image-guided intra-articular corticosteroid injection for hip OA. Seventy-five patients were injected with 40 mg of methylprednisolone and 45 patients were injected with 80 mg. For the group with the 40 mg dose there was a statistically significant improvement in pain and stiffness but not disability as assessed by Western Ontario and McMaster Universities Osteoarthritis Index (WOMAC) at 6 weeks, and only the improvement in stiffness at 12 weeks was maintained. For the 80 mg dose, there was a significant improvement in pain, stiffness and disability at 6 weeks, which was maintained at 12 weeks. When the doses were compared, the 80 mg dose demonstrated a significant improvement compared with the 40 mg group for stiffness at Week 12 and disability at both Weeks 6 and 12. Imaging findings did not correlate to severity of symptoms or response to injection. The authors concluded that both the 40 mg and 80 mg doses had a beneficial effect at Week 6, whereas the 80 mg dose maintained this improvement at Week 12.

Young *et al*. [50] investigated whether volume was important in therapeutic hip injections. They conducted a randomized trial of 110 patients with hip OA who received 40 mg triamcinolone and 2 ml bupivacaine with or without addition of 6 ml of sterile water. They reported no difference between two groups in pain symptoms measured at two weekly intervals of WOMAC scores at 3 months.

All the above studies pertain to the rheumatology literature. There are no specific studies in the orthopaedic literature that have evaluated the efficacy of intra-articular corticosteroids on hip OA. The only study of relevance is by Kaspar *et al*. [44] who conducted a survey of 99 orthopaedic hip surgeons in Ontario on their use of steroid hip injections for OA. They reported that 56% of surgeons found that steroid hip injections were clinically useful, 25% believed it may accelerate arthritis and 19% believed it may increase infection of subsequent arthroplasty. However, much of the expert opinion in this survey was not based on evidence from the literature but more on anecdotal experiences.

### Local risks of corticosteroid intra-articular hip injections

The local risks of intra-articular corticosteroid injections included skin dislocation, fatty atrophy, exacerbation of pain, septic arthritis and a potential increased risk in infection risk of any subsequent arthroplasty procedure [47, 51–53]. There have been case reports of septic arthritis following intra-articular steroid injections [54–56]. These mainly involve skin organisms. With respect to increased rates of infection with subsequent arthroplasty, Meermans *et al*. [57] performed a retrospective review of 175 patients who underwent THA following a steroid injection and reported no increased risk of infection. Similarly, McMahon *et al*. [58] reported no increase in infection rates in 49 patients with THA up to 8 years following a steroid injection. Wang *et al*. [59] conducted a meta-analysis of 11 retrospective case series of infection rates in total knee and THA in patients who has received intra-articular steroids. They reported on 1474 participants with 14 deep and 72 superficial infections. The relative risk of infection for subsequent arthroplasty was not increased with intra-articular steroid.

## HA INTRA-ARTICULAR HIP INJECTIONS

### The efficacy of HA intra-articular hip injections for pain relief for OA of the hip

HA is a glycosaminoglycan found in connective tissue, epithelial tissue, neural tissue and synovial fluid [60–63]. There are mixed results in the literature regarding the efficacy of intra-articular HA in the treatment of pain from OA of the hip. Richette *et al*. [64] conducted a multicenter, randomized, parallel-group, placebo-controlled trial to evaluate the efficacy and tolerability of a single intra-articular injection of HA (2.5 ml) under fluoroscopic guidance for the treatment of hip OA. Forty-two patients received HA and 43 patients received a placebo. At 3 months, the decrease in pain score as measured by the visual analogue scale (VAS) did not differ between the groups with the responder rates being 33.3% and 32.6% respectively. Other secondary endpoints as measured by the WOMAC score did not differ between the two groups. The authors concluded that a single intra-articular injection of HA is no more effective than placebo in treating the symptoms of hip OA.

In contrast to the above findings, Migliore *et al*. [65] conducted a prospective double-blinded randomized control trial of 42 patients with hip OA who either received 1500–2000 kDa of HA or mepivacaine administered twice once a month under ultrasound guidance. Patients in the HA group exhibited a significantly reduced Lequesne's algofunctional index and VAS pain scores three and 6 months after treatment compared with the local anaesthetic group. The authors concluded a beneficial effect of intra-articular HA in the management of hip OA. In another study, Migliore *et al*. [66] reported that ultrasound guided HA injections in 2,343 patients reduced the consumption of non-steroidal anti-inflammatory medication by 48.2% at 3 months, 50% at 12 months and 61% at 24 months.

With respect to the dosage of intra-articular, HA Tikiz *et al*. [67] randomized 43 patients to receive either low molecular weight HA or high molecular weight HA injections weekly for 6 months. They reported an improvement in outcome and pain scores at 1 month, which was maintained at 6 months but demonstrated no difference between the groups. There was a 9% and 12% incidence of local pain and swelling at injection site for high molecular weight and low molecular weight groups respectively.

There have been several studies that have compared the efficacy of intra-articular corticosteroid with HA in the management of OA of the hip. Atchia *et al*. randomized 77 patients to either receive a single ultrasound guided intra-articular injection of normal saline, HA or methylprednisolone [68]. They found that pain and functional scores improved in the corticosteroid arm at 1 week and was maintained over 8 weeks. There was no difference in scores between the HA and normal saline groups. Qvistgaard *et al*. [69] reported similar findings. They randomized 101 patients to receive three ultrasound guided intra-articular injections of either HA, corticosteroid or normal saline. The corticosteroid arm produced the most significant improvement of pain on weight bearing at 3 months based on the VAS. HA produced a smaller but still significant improvement.

This is in contrast to the findings of Spitzer *et al*. [70] who conducted a prospective randomized study of 313 patients approximately half of which received intra-articular HA (Hylan G-F 20) 2 weeks apart or one injection of 40 mg of methylprednisolone and one sham injection 2 weeks apart. At 6 months functional scores for Hylan G-F 20 were higher than methylprednisolone for patients with more severe OA (Kellgren Lawrence Grade 3) and similar for less severe OA (Kellgran Lawrence Grade 2) [70].

## PRP INTRA-ARTICULAR HIP INJECTIONS

### The efficacy of PRP intra-articular hip injections for pain relief for OA of the hip

The efficacy of PRP intra-articular injections in management of OA of the hip has been less extensively studied compared with corticosteroids and HA. PRP has shown efficacy in cases series involving OA of the knee but there is less literature on its effects on hip OA [71]. Sanchez *et al*. [72] conducted a non-controlled prospective study on 40 patients with OA of the hip who received three injections of PRP once a week. Statistically significant reductions in VAS, WOMAC and Harris hip subscores (HHS) for pain and function were reported at 7 weeks and 6 months. The authors concluded that PRP was effective for the management of pain from OA of the hip. However, the criteria for significance defined in the study was 30% reduction in pain and disability, which from the results of Richette *et al* [64] can also be achieved with a placebo.

Battaglia *et al*. [73] conducted a randomized trial comparing the efficacy of HA to PRP on the management of pain associated with OA of the hip. One hundred patients were randomly assigned to receive an ultrasound guided intra-articular injection of either PRP or HA. They found that Intra-articular injections of PRP are efficacious in terms of functional improvement and pain reduction but are not superior to HA in patients with symptomatic hip OA at 1-, 3-, 6- and 12-month follow-up with VAS and Harris Hip Scores. Table II summaries the role of biologics in the management of OA of the hip.

**Table II.** Summary of the use of intra-articular biologics in management of OA of the hip

	Corticosteroids	HA	PRP
Level of evidence	Randomized controlled double blinded trials	Randomized controlled blinded trials Randomized trials of corticosteroids versus HA Randomized trial of HA versus PRP	Non-controlled prospective study
Efficacy	Significant reduction in pain and improvement in hip scores for up to 12 weeks 80 mg MPL produces a sustained improvement in pain, stiffness and function compared with 40 mg MPL Volume of injection does not affect efficacy	Discrepancy regarding efficacy with some level 1 trials showing no difference compared with placebo and others a significant reduction in pain and improvement in hip scores Increased doses do not improve efficacy More trials suggest reduced efficacy compared with CS HA superior reduction in pain and improvement in hip scores compared with PRP	Significant reduction in pain and improvement in hip scores at 7 weeks and 6 months
Side Effects	Local reaction septic arthritis Infection post arthroplasty reported but no evidence to suggest infection rate of subsequent arthroplasty is increased	9–12% incidence of pain at injection site and redness	None reported

### The role of intra-articular hip injections in FAI and labral tears

There is a paucity of literature on the efficacy of intra-articular biologics in the management of labral tears and FAI in comparison to OA of the hip. Krych *et al*. [74] reported on the results of corticosteroid intra-articular injections in a cohort of 54 patients with labral tears and FAI. The found that at 14 days post-injection, only 20 patients (37 %) and at 6 weeks only three patients (6 %) reported a clinically significant decrease in pain. The average duration of pain relief was 9.8 days. There was no difference in pain reduction between steroid preparations. The authors concluded that in patients with symptomatic FAI and labral tear, intra-articular cortisone injection has limited clinical benefit as a therapeutic modality. Nevertheless, anaesthetic-only intra-articular injections for patients who may be candidates for hip arthroscopy can be a useful diagnostic tool [75–77].

Abate *et al*.[78] examined the effects of intra-articular HA on 23 patients with FAI. They reported a significant reduction in pain scores, Lequesne index and non-steroidal anti-inflammatory consumption after 6 and 12 months compared with baseline. Mean HHS scores also significantly improved from baseline from this time period. The study was not controlled and may therefore be potentially affected by investigator and patient bias. Nonetheless, the results suggest a potential role for intra-articular HA in the non-operative management of FAI.

Redmond *et al*. [79] evaluated the effect of intraoperative PRP injection on the outcomes of patients undergoing hip arthroscopy for labral treatment. Ninety-one patients received PRP and 180 patients received 0.25% bupivacaine. The patients that received PRP had slightly higher but statistically significant pain scores and slightly lower modified HSS (mHHS) at 2-year follow-up. The authors concluded that intra-operative PRP injection does not appear to improve the clinical results of patients undergoing hip arthroscopy for labral treatment. Table III summaries the role of biologics in the management of FAI and labral tears.

**Table III.** Summary of the use of intra-articular biologics in the management of FAI and labral tears of the hip

	Corticosteroids	HA	PRP
Level of evidence	Non-randomized, non controlled prospective study	Non-randomized, non controlled prospective study	Controlled randomized prospective study
Efficacy	No improvement in pain	Improvement in pain, NSAID consumption and hip scores at 6 and 12 months compared with baseline	Intra-operative PRP does not improve clinical results of arthroscopic labral treatment
Side effects	None reported	Injection site pain and redness	None reported

## CONCLUSION

Intra-articular hip injections have both a therapeutic and diagnostic utility in the management of hip disorders. Intra-articular local anaesthetic injections, especially with image guidance, can help delineate an intra-articular versus extra-articular versus spinal source of hip symptoms and has a high positive predictive value for successful surgery. With respect to intra-articular hip biologics, the most widely studied medications are corticosteroids, HA and PRP. In the setting of OA, corticosteroids appear to be the most effective, providing significant pain relief for up to 12 weeks. Eighty milligrams of methylprednisolone are more effective than 40 mg in providing sustained pain relief. Intra-articular corticosteroids do not seem to increase the risk of infection in subsequent arthroplasty procedures In the setting of FAI and labral tears corticosteroids have little therapeutic benefit and should not be routinely used especially considering the in-vitro risk of chondrotoxicity. The efficacy of HA intra-articular injections has had mixed results in the literature. It appears that they may be more effective than PRP but less effective than corticosteroids. HA and PRP may have a role in treatment of OA in patients who can not receive intra-articular steroids. There is some weak evidence to suggest that HA may be beneficial in reducing pain associated with labral tears but currently there is no evidence to warrant the routine use of therapeutic intra-articular injections in labral tears and FAI. In this population injections should be limited to diagnostic purposes.

## FUNDING

This work was supported by the American Hip Institute.

## CONFLICT OF INTEREST STATEMENT

Dr. Domb reports research support from American Hip Institute, Arthrex, Inc., MAKO Surgical Corp., Breg, ATI and Pacira. Dr. Domb receives consulting fees from Arthrex, Inc., MAKO Surgical Corp., and Pacira. Dr. Domb owns stock in Stryker, and receives royalties from Arthrex, Inc., Orthomerica and DJO Global. He is also a member of the AANA Learning Center Committee.
